# Application of a self-developed femoral artery compression hemostasis device in proximal femoral nail anti-rotation surgery for intertrochanteric fractures: a case report

**DOI:** 10.3389/fsurg.2026.1871267

**Published:** 2026-06-03

**Authors:** Limin He, Tietao Di, Dandan Wang, Qihong Wu, Bin Zhao, Po Yang

**Affiliations:** 1 Department of Orthopedic Trauma, The Second Affiliated Hospital of Guizhou University of Traditional Chinese Medicine, Guiyang, Guizhou, China; 2 Department of Orthopedic Trauma, The Fourth People's Hospital of Guiyang, Guiyang, Guizhou, China

**Keywords:** femoral artery compression, hemostatic device, intertrochanteric femoral fracture, intraoperative blood loss, ischemia-reperfusion injury, PFNA

## Abstract

**Background:**

Intertrochanteric fractures of the femur are a prevalent type of hip fracture among the elderly. While surgical treatment is the preferred approach for managing these fractures, conventional tourniquets cannot be applied in hip surgery due to anatomical constraints. As a result, effectively controlling intraoperative and perioperative blood loss has long posed a significant clinical challenge.

**Case presentation:**

A 70-year-old male patient with a comminuted fracture in the left intertrochanteric region of the femur underwent closed reduction and internal fixation using a proximal femoral nail anti-rotation (PFNA). During the procedure, a proprietary compression-type femoral artery hemostasis device was used to effectively control regional blood flow. The femoral artery pulsation was palpated inferior to the inguinal ligament and superior to the pubic ramus. A compression device was then applied to achieve precise compression of the femoral artery. Effective compression was indicated by the absence of the dorsalis pedis artery pulse, which helped maintain collateral circulation. The procedure lasted 45 min, with an estimated intraoperative visible blood loss of 50 mL. Quantitative analysis using the Gross formula, based on the patient's hematocrit decline from 0.394 preoperatively to 0.358 on postoperative day 2, revealed a calculated total perioperative blood loss of 433 mL, with hidden blood loss accounting for 383 mL. There were no postoperative complications, including limb ischemia, nerve injury, or deep vein thrombosis. The patient was discharged in good condition 6 days after surgery. A follow-up examination at 6 weeks postoperatively revealed satisfactory callus formation at the fracture site and stable internal fixation.

**Conclusion:**

In this single case, application of the self-developed femoral artery compression hemostatic device was associated with reduced intraoperative blood loss and a favorable safety profile during PFNA fixation of an intertrochanteric fracture. The device may offer a novel, non-invasive hemostatic strategy for selected hip fracture surgeries; however, further studies are required to confirm its efficacy and safety.

## 1 Introduction

Intertrochanteric fractures of the femur are prevalent among the elderly, leading to significant incidence rates and increased disability. Surgical reduction and internal fixation facilitate early ambulation and help minimize complications associated with extended periods of bed rest (1). Currently, intramedullary fixation methods (such as PFNA) are the standard surgical approach. These techniques provide secure fixation while minimizing trauma to surrounding tissues (2). However, due to the location of the incision at the proximal end of the limb during hip surgery, conventional inflatable tourniquets cannot be utilized. Intraoperative hemostasis primarily relies on methods such as electrocoagulation, bone wax, and medications, which are often ineffective. This reliance can lead to excessive intraoperative bleeding and significant postoperative occult blood loss (3).

Current hemostatic strategies for hip fracture surgery include mechanical, pharmacological, and local hemostatic approaches. Conventional tourniquets are effective for distal limb surgery but cannot be used at the hip due to anatomical constraints and risk of ischemia–reperfusion injury. Topical hemostatic agents (e.g., bone wax, fibrin glue) are limited to small bone or soft-tissue bleeding and do not affect major arterial inflow. Systemic tranexamic acid (TXA) reduces overall blood loss but is contraindicated in some high-risk patients. Vascular closure devices are designed for percutaneous puncture sites and are not suitable for open orthopedic procedures. Given these limitations, a safe, non-invasive, and controllable method to reduce proximal arterial inflow during hip surgery remains an unmet clinical need.

Tranexamic acid (TXA) has been widely shown to reduce perioperative blood loss in hip fracture surgery (4, 5). High-quality evidence, including recent meta-analyses, demonstrates that TXA is generally safe in this setting and does not significantly increase the risk of thromboembolic events in most elderly patients (6). However, in patients with absolute contraindications to TXA (e.g., active thromboembolic disease, certain anticoagulation regimens, or high thromboembolic risk), alternative hemostatic strategies are needed. Mechanical femoral artery compression may serve as a valuable non-pharmacological alternative in these carefully selected patients.

Traditional tourniquets control bleeding by completely occluding both arterial and venous blood flow in the limb; however, they have a significant drawback: Prolonged complete ischemia can easily result in muscle and nerve damage. Ischemia-reperfusion injury may occur upon tourniquet release. They cannot be applied to areas close to the trunk, such as the hip. The simultaneous occlusion of both arteries and veins can exacerbate venous bleeding if compression is not complete (7–9).

The femoral artery drives blood to the lower limbs. The hip and proximal femur have a rich collateral arterial network, which is crucial for maintaining perfusion when the main femoral artery is temporarily compressed. This network includes the superior gluteal artery, inferior gluteal artery, medial femoral circumflex artery, lateral femoral circumflex artery, and profunda femoris artery, forming extensive anastomoses around the hip joint. These collaterals ensure adequate blood supply to the proximal femur and hip musculature even when the main femoral artery trunk is occluded, which provides the anatomical basis for our “trunk occlusion with collateral preservation” hemostasis strategy. A recent anatomical study further confirmed the complexity and consistency of this collateral system, emphasizing its clinical significance in hip fracture surgery (10). Building on this anatomical knowledge, our team developed the “compression-type Femoral Artery Hemostasis Device” (Patent No: ZL 2020 2 0249524.5). This device enables regional hemostasis via “trunk occlusion with collateral preservation,” effectively reducing bleeding at the surgical site while preserving blood flow to distal tissues. This innovative approach helps prevent complications related to complete ischemia (11). This case report describes the clinical application of the device during PFNA fixation and evaluates its feasibility and safety profile in a single patient.

## 2 Case report

### 2.1 Patient information

A 70-year-old male patient was admitted to the hospital 3 h after slipping at a swimming pool, exhibiting left hip pain and restricted mobility. The patient underwent an appendectomy at age 50 for acute appendicitis and had internal fixation for a patellar fracture 10 years ago. He has no history of chronic conditions, including hypertension, diabetes, or coronary artery disease.

Physical examination revealed tenderness, swelling, and subcutaneous ecchymosis in the left hip. There was positive longitudinal percussion tenderness in the left lower limb, along with limited mobility and approximately 1 cm of shortening compared to the contralateral limb. The dorsalis pedis artery pulse was palpable on the left side, and skin sensation, perfusion, and temperature were normal.

**Figure 1:**
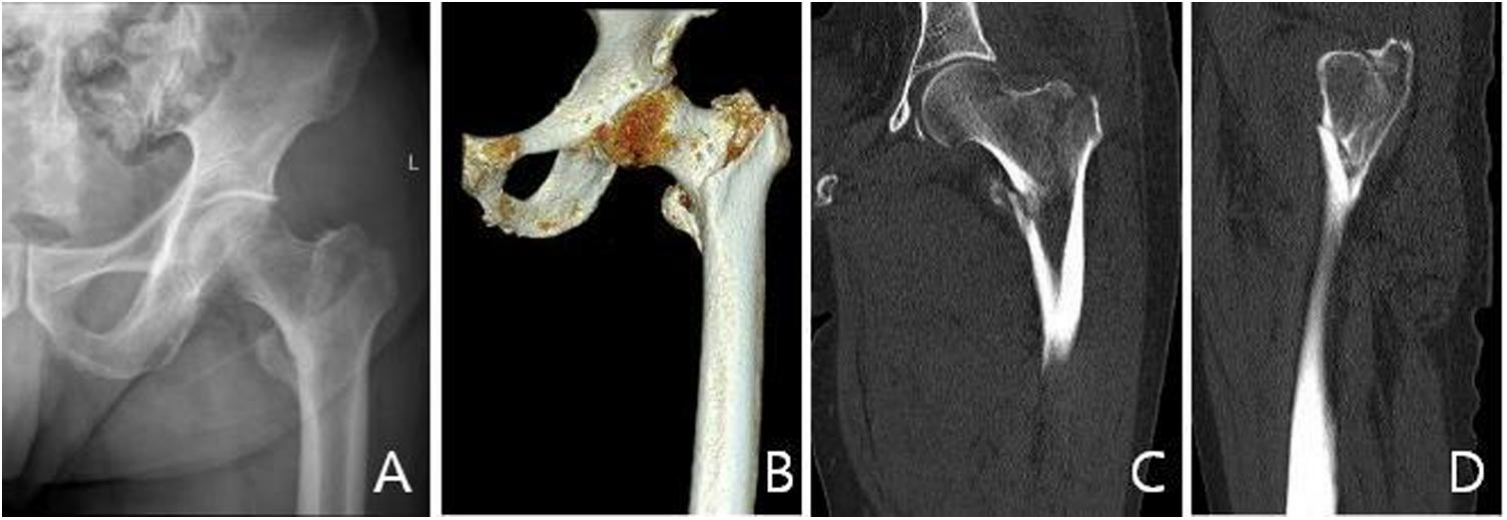
Preoperative radiographs and CT. **(A)** preoperative anteroposterior X-ray images of the left hip. **(B)** Three-dimensional CT reconstruction. **(C)** Coronal CT image. **(D)** Sagittal CT image.

The patient weighed 75 kg and was 160 cm in height. Laboratory findings on admission revealed a hemoglobin level of 134 g/L, a hematocrit of 0.394 L/L, a white blood cell count of 12.32 × 10^9^/L, and a D-dimer level of 6.010 μg/mL FEU. Imaging Studies: Anteroposterior radiograph of the pelvis shows an intertrochanteric fracture of the left femur with slight displacement of the fracture ends (Figure 1A). Three-dimensional reconstructed computed tomography (CT) shows a comminuted intertrochanteric fracture of the left femur with varus angulation deformity of the fracture ends (Figures 1B–D).

### 2.2 Diagnosis and treatment options

The preoperative diagnosis for the patient was a comminuted intertrochanteric fracture of the left femur. Treatment Plan: Closed reduction followed by PFNA internal fixation under spinal anesthesia, with intraoperative bleeding controlled using a proprietary femoral artery compression hemostatic device. This study received approval from the hospital's Institutional Review Board, and informed consent was obtained from the patient and their family members. The treatment timeline is outlined in Table 1.

**Table 1:** Timeline of the treatment process for the reported case.

Time point	Key events	Findings/Actions
Day 0 (Injury) −1	Slipped in the swimming pool	Severe hip pain with inability to bear weight; x-ray examination at our hospital confirmed a fracture; the patient was admitted for further diagnostic evaluations
Day 1–3	Preoperative Planning	Evaluation, anticoagulation; skin preparation; adjustment of hemostatic devices
Day 4	surgery	Perform PFNA internal fixation with femoral artery compression to control bleeding.
Postoperative Days 1–5	Rehabilitation	Bed rest; Deep vein thrombosis (DVT) prevention;Wound dressing changes; Follow-up complete blood count (CBC) and x-ray; Functional rehabilitation exercises
Postoperative Day 6	Rehabilitation	The wound was well-managed, and the patient was discharged without issues.
Postoperative Week 6	Follow-up visit	The outpatient follow-up x-ray indicated callus formation, and partial weight-bearing was noted.

### 2.3 Surgical procedures and device utilization

#### 2.3.1 Anesthesia and positioning

**Figure 2:**
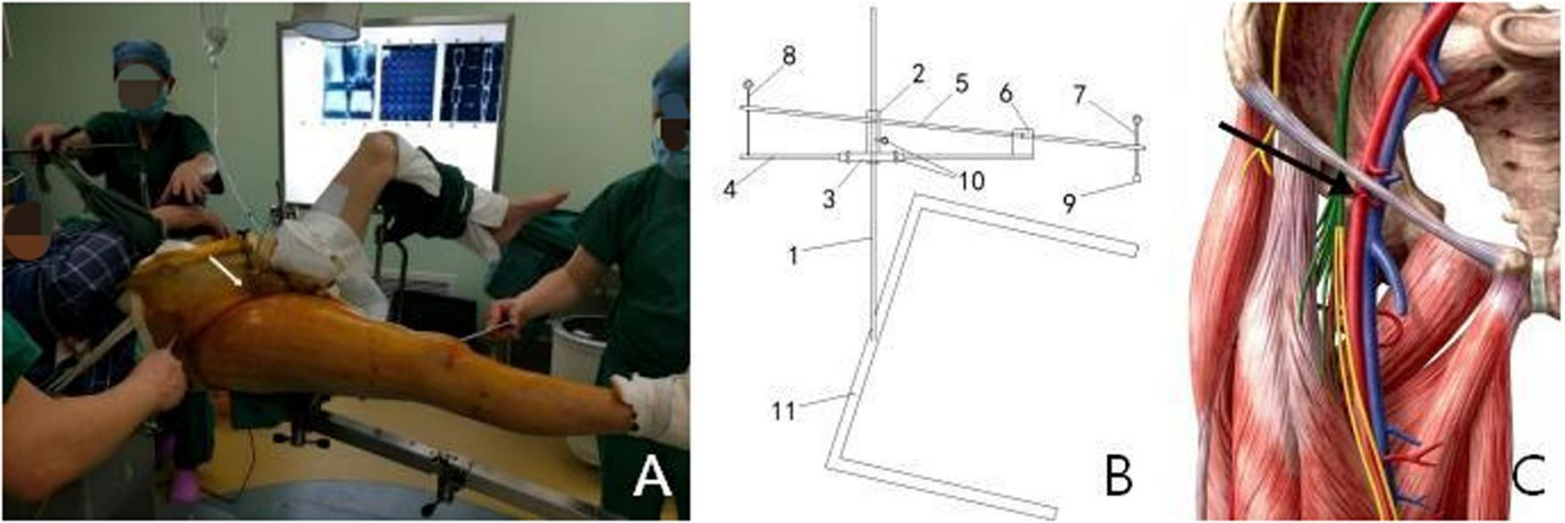
**(A)** In clinical specimens, the white arrow marks where compression is applied. **(B)** Schematic diagram of the structure of a femoral artery compression hemostasis device. **(C)** In the regional anatomical schematic, the position marked by the black arrow indicates the compression site. (Patient's facial features are pixelated to protect confidentiality.)

Once spinal anesthesia has been administered, the patient was positioned supine on the orthopedic traction table. The unaffected limb is abducted while the affected limb is placed in a neutral position to facilitate traction reduction (Figure 2A).

#### 2.3.2 Structure and function of hemostatic devices

The self-developed compression-type femoral artery hemostasis device consists of four main components: (1) a pressure pad “9” made of soft, non-slip silicone (1 cm × 3 cm), designed to conform to the femoral artery pulsation area; (2) an adjustable compression rod “7”, a 10 cm stainless steel rod connected to the pressure pad via a ball joint for multi-angle adjustment; (3) a precision adjustment screw “8”, with a threaded design enabling 1 mm incremental adjustment of compression force; (4) a rigid fixed base “11”, curved to match the pelvic contour with a non-slip bottom surface for stable placement during surgery. The device provides sustained, adjustable compression when positioned correctly (Figure 2B). Compression Site: Below the inguinal ligament, above the pubic branch, and at the location of femoral artery pulsation. Mechanically, the device uses the pubic ramus as a solid anchor and presses directly on the femoral artery trunk just distal to the inguinal ligament (Figure 2C). Intervention Target: To selectively occlude the main femoral artery while maintaining collateral circulation, thus preventing complete distal ischemia.

#### 2.3.3 Instructions for using hemostatic devices

(1) Palpate the inguinal region to locate the strongest pulsation of the femoral artery.
(2) Position the hemostatic device, ensuring the compression pad “9” is aligned with the femoral artery pulsation point.
(3) Slowly turn the adjustment screw “8” to apply pressure until the pulse of the dorsalis pedis artery on the same side disappears.
(4) Maintain this compression until the fracture surgery is complete.
(5) Gradually release the pressure and monitor the recovery of blood supply, color, and temperature in the foot.

#### 2.3.4 Surgical technique

After successful closed reduction via orthopedic traction and confirmation by fluoroscopy, routine disinfection and draping were performed. A 5 cm vertical incision was made at the lateral aspect of the greater trochanter. The fascia lata was incised, and the tip of the greater trochanter was exposed. A guide pin was inserted at the anterior 1/3 and posterior 2/3 junction of the greater trochanter under fluoroscopic guidance. The medullary canal was opened with a reamer, and the PFNA main nail was inserted along the guide pin. A helical blade was then inserted into the femoral head through a lateral aiming device, with the tip positioned 5–10 mm subchondral to the femoral articular surface. Distal locking screws were placed under fluoroscopy, and the locking cap was secured. The femoral artery compression device was kept in place throughout the entire procedure to ensure there was no active vascular bleeding in the surgical area.

### 2.4 Follow-up and outcomes

**Figure 3:**
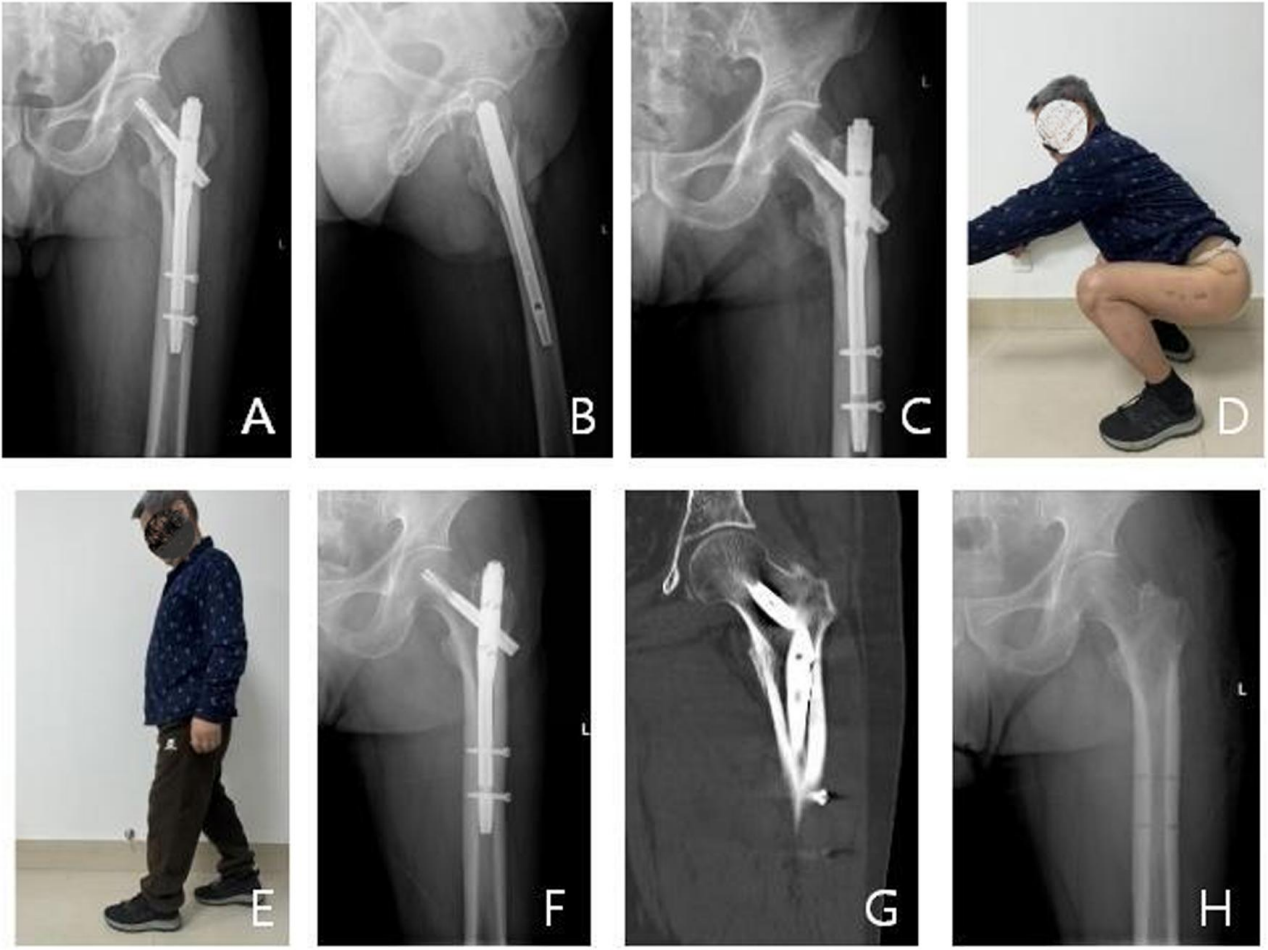
Postoperative follow-up imaging. **(A)** Anteroposterior radiograph of the left femur on postoperative day 2. **(B)** Lateral radiograph of the left femur on postoperative day 2. **(C)** Anteroposterior radiograph of the left hip at 6 weeks postoperatively. **(D)** Clinical photograph showing hip flexion at 6 months postoperatively. **(E)** Clinical photograph demonstrating standing and walking posture at 6 months postoperatively. **(F)** Radiograph obtained at 1 year postoperatively showing healed fracture site. **(G)** Coronal computed tomography (CT) image of the left femur at 1 year postoperatively. **(H)** Radiograph obtained 1 year postoperatively after removal of the internal fixation device. (Patient's facial features are pixelated to protect confidentiality.)

The total operative time was 45 min, with an estimated intraoperative blood loss of 50 mL. No blood transfusion or hemostatic agents were administered. Postoperative radiographs demonstrated satisfactory fracture reduction with appropriate implant positioning and no evidence of helical blade penetration into the articular surface (Figures 3A, B). On postoperative day 2, the patient was able to sit upright in bed without significant pain. To precisely quantify perioperative blood loss, the patient's total blood volume was first calculated using the Nadler formula [total blood volume [L] = 0.3669 × height^3^ [m] + 0.03219 × weight [kg] + 0.6041 for male adults] (12). We calculated a total blood volume of 4,521 mL based on a body weight of 75 kg and height of 160 cm. Applying the Gross formula, the perioperative total blood loss—derived from the decline in hematocrit from 0.394 L/L preoperatively to 0.358 L/L on postoperative day 2—was 433 mL. Of this, hidden blood loss accounted for 383 mL, whereas intraoperative visible blood loss was a mere 50 mL. It is noteworthy that the calculated total blood loss of 433 mL lies at the lower margin of the 400–760 mL range typically reported in the literature for intertrochanteric fracture fixation, and the absolute hidden blood loss volume was substantially less than commonly observed (13). However, this comparison with pooled literature data should be interpreted cautiously, given the inherent limitations of a single case observation. Patient-specific variables and intraoperative hemodynamic management may have contributed to this favorable outcome. Laboratory tests on the same day showed a hemoglobin level of 108 g/L, a hematocrit of 0.358 L/L, a white blood cell count of 12.29 × 10^9^/L, and a D-dimer level of 2.720 μg/mL FEU.

On postoperative day 6, the wound presented no redness, exudate, or signs of limb ischemia, nerve injury, or deep vein thrombosis, and the patient was discharged uneventfully. At the 6-week follow-up, radiographs demonstrated robust callus formation at the fracture site, with stable internal fixation and no implant breakage (Figure 3C). At the six-month follow-up, the patient had regained independent ambulation and proficiency in performing daily activities, including full flexion of the hip and knee joints (Figures 3D, E). The Harris Hip Score was 90 points at six months, marking strong hip recovery. One year after surgery, both x-ray and CT scans confirmed complete fracture healing, with the fixation remaining stable (Figures 3F, G). The patient subsequently underwent elective readmission for removal of the internal fixation hardware and was discharged with satisfactory outcomes (Figure 3H).

### 2.5 Patient perspective

During follow-up, the patient's pain was gradually relieved, and physical activity recovered satisfactorily. The patient was satisfied with the surgical trauma, blood loss, pain management, and recovery speed. No device-related adverse events associated with hemostasis were observed.

## 3 Discussion

The perioperative total blood loss associated with intertrochanteric femoral fractures is substantial, typically ranging from 400 to 760 mL, with hidden blood loss representing over 70%–85% of this volume (13). Even with minimally invasive PFNA techniques, blood loss can precipitate anemia, prolong hospitalization, and increase mortality in elderly individuals (3). The anatomical infeasibility of applying a conventional tourniquet at the hip renders hemostatic management uniquely challenging in orthopedic trauma surgery (7).

Current conventional hemostatic measures have noteworthy limitations. Although systemic or topical TXA effectively reduces blood loss in hip fracture surgery and is generally safe per current evidence, its use may be contraindicated in specific high-risk subgroups, such as patients on therapeutic anticoagulation or with a history of recent thromboembolism. In these cases, mechanical hemostasis offers a promising alternative (3, 14). Intraoperative modalities such as electrocautery and bone wax are only effective for controlling minor soft-tissue or bone-surface bleeding, whereas gauze packing provides only temporary hemostasis and interferes with surgical manipulation (15). Vascular closure devices are designed primarily for percutaneous interventional sites and are unsuitable for open or minimally invasive orthopedic procedures (16, 17). Conventional tourniquets, which render the entire limb ischemic, carry well-documented risks of ischemia-reperfusion injury, nerve palsy, and muscle necrosis (8, 9).

The present device is designed based on the distinct collateral vascular anatomy of the hip and proximal femur (10). By selectively compressing only the femoral arterial trunk, it leaves the collateral pathways intact, thereby achieving a precision hemostatic model defined as “truncal occlusion with collateral preservation”. This mechanism confers several core advantages. First, it occludes only the main arterial channel while preserving low-flow distal perfusion via collaterals, thus mitigating the risks of complete tissue ischemia, systemic release of metabolic byproducts, and ischemia-reperfusion injury (11, 18). Second, the device employs the pubic ramus as a rigid counterforce fulcrum, providing fixed-point compression with uniform pressure distribution that remains stable despite limb manipulation. Third, the compression endpoint—ablation of the dorsalis pedis pulse—is an objective, readily assessable bedside parameter that enables individualized pressure titration, thereby minimizing the risk of excessive compression and potential intimal injury. Importantly, many elderly hip fracture patients receive long-term anticoagulation or have elevated thromboembolic risk, making TXA relatively contraindicated. In these patients, mechanical femoral artery compression may offer a useful non-pharmacological alternative for hemostasis. Fourth, the device's inguinal positioning is entirely remote from the lateral surgical approach, ensuring no interference with the standard minimally invasive PFNA workflow. Fifth, its applicability can extend beyond PFNA to encompass dynamic hip screw fixation, arthroplasty, and even pre-hospital control of junctional hemorrhage (19).

However, it is critical to acknowledge that selective compression of the femoral artery trunk inherently carries potential risks of endothelial injury, thrombosis, or pseudoaneurysm (20, 21). A single uneventful case cannot establish safety. The absence of vascular complications in this patient, while encouraging, must be interpreted with caution. Future prospective investigations should incorporate routine postoperative vascular ultrasound to evaluate vessel patency and wall integrity objectively, and systematically document any device-related adverse events in larger cohorts. In this case, the 45-minute compression time was well tolerated, with no ischemic complications, demonstrating that short-term, selective femoral artery compression with preserved collateral perfusion and gradual decompression has a favorable safety profile. These attributes align closely with Enhanced Recovery After Surgery (ERAS) principles (22), offering a cost-effective strategy to reduce transfusion requirements and expedite functional recovery.

The present study has several important limitations that must be acknowledged. First, this is a single-case report with no control group, and no formal statistical analysis can be performed, limiting the generalizability of the findings. Second, no objective vascular assessment was performed, including Doppler ultrasound, ankle–brachial index (ABI) measurement, or postoperative vascular imaging. Arterial occlusion during compression was judged solely by clinical palpation of the dorsalis pedis artery pulse; no objective confirmation of arterial flow reduction or occlusion was obtained, and the proposed hemostatic mechanism remains theoretically inferred rather than empirically validated. Third, the optimal compression pressure, safe compression duration, and long-term vascular sequelae remain undetermined. Fourth, the device is a self-developed prototype with a Chinese utility model patent (ZL 2020 2 0249524.5). It has not yet completed formal medical device registration, standardized preclinical safety testing, or prospective clinical trial registration. The present use was an exploratory clinical application approved by the institutional ethics committee. The absence of a comparative cohort prevents definitive evaluation of therapeutic superiority. Key parameters remain undetermined, including the optimal compression pressure, the maximum safe duration of compression, and long-term vascular sequelae, underscoring the need for further biomechanical and prospective clinical studies to elucidate these factors.

Beyond hip fracture surgery, controlled femoral artery compression may also have potential utility in managing acute femoral artery hemorrhage in trauma (e.g., butcher's knife injuries, stab wounds) or other injuries where the artery is superficial within the femoral triangle. Further research is needed to explore these applications.

## 4 Conclusion

In this case report, the self-developed femoral artery compression hemostatic device was feasible and well tolerated during PFNA fixation of an intertrochanteric fracture. Intraoperative blood loss was low, and no device-related complications were observed. The device was easy to apply and did not disrupt the standard surgical workflow. The concept of “truncal occlusion with collateral preservation” represents a potential novel, non-invasive hemostatic strategy for selected hip fracture procedures. However, given the limitations of a single case, no definitive conclusions regarding general efficacy or safety can be drawn. Larger clinical studies are necessary to validate these findings.

## Data Availability

The original contributions presented in the study are included in the article/Supplementary Material, further inquiries can be directed to the corresponding authors.
